# Apigenin blocks IKKα activation and suppresses prostate cancer progression

**DOI:** 10.18632/oncotarget.5157

**Published:** 2015-09-05

**Authors:** Sanjeev Shukla, Rajnee Kanwal, Eswar Shankar, Manish Datt, Mark R. Chance, Pingfu Fu, Gregory T. MacLennan, Sanjay Gupta

**Affiliations:** ^1^ Department of Urology, Case Western Reserve University & University Hospitals Case Medical Center, Cleveland, Ohio 44106, USA; ^2^ The Urology Institute, University Hospitals Case Medical Center, Cleveland, Ohio 44106, USA; ^3^ Center for Proteomics and Bioinformatics, Case Western Reserve University, Cleveland, Ohio 44106, USA; ^4^ Department of Epidemiology & Biostatistics, Case Western Reserve University & University Hospitals Case Medical Center, Cleveland, Ohio 44106, USA; ^5^ Department of Pathology, Case Western Reserve University & University Hospitals Case Medical Center, Cleveland, Ohio 44106, USA; ^6^ Department of Nutrition, Case Western Reserve University & University Hospitals Case Medical Center, Cleveland, Ohio 44106, USA; ^7^ Divison of General Medical Sciences, Case Comprehensive Cancer Center, Cleveland, Ohio 44106, USA; ^8^ Department of Urology, Louis Stokes Cleveland Veterans Affairs Medical Center, Cleveland, Ohio 44106, USA

**Keywords:** prostate cancer, apigenin, NF-ĸB signaling, therapeutic target, cell cycle

## Abstract

IKKα has been implicated as a key regulator of oncogenesis and driver of the metastatic process; therefore is regarded as a promising therapeutic target in anticancer drug development. In spite of the progress made in the development of IKK inhibitors, no potent IKKα inhibitor(s) have been identified. Our multistep approach of molecular modeling and direct binding has led to the identification of plant flavone apigenin as a specific IKKα inhibitor. Here we report apigenin, in micro molar range, inhibits IKKα kinase activity, demonstrates anti-proliferative and anti-invasive activities in functional cell based assays and exhibits anticancer efficacy in experimental tumor model. We found that apigenin directly binds with IKKα, attenuates IKKα kinase activity and suppresses NF-ĸB/p65 activation in human prostate cancer PC-3 and 22Rv1 cells much more effectively than IKK inhibitor, PS1145. We also showed that apigenin caused cell cycle arrest similar to knockdown of IKKα in prostate cancer cells. Studies in xenograft mouse model indicate that apigenin feeding suppresses tumor growth, lowers proliferation and enhances apoptosis. These effects correlated with inhibition of *p*-IKKα, NF-ĸB/p65, proliferating cell nuclear antigen and increase in cleaved caspase 3 expression in a dose-dependent manner. Overall, our results suggest that inhibition of cell proliferation, invasiveness and decrease in tumor growth by apigenin are mediated by its ability to suppress IKKα and downstream targets affecting NF-ĸB signaling pathways.

## INTRODUCTION

The IĸB kinases IKKα and IKKβ are critical in activating the NF-ĸB pathway [1, 2]. IKKα/β are catalytic subunits of the heterotrimeric IKK complex bound to the non-catalytic subunit IKKγ/Nemo [1–4]. IKKα/β phosphorylates amino terminal serine residues in the IĸB family proteins. Phosphorylation of IĸB leads to ubiquitination and subsequent degradation by the proteasome, resulting in the nuclear translocation of NF-ĸB (p65/p50), designated as the canonical pathway [5–7]. An alternative pathway, regulated by IKKα homodimers, induces processing of the precursor p100 to NF-ĸB2 (p52) to translocate with RelB to the nucleus, leading to DNA binding and target gene activation [5–7].

Aberrant activation of IKKs, NF-ĸB subunits and their regulated pathways have been implicated in the pathogenesis of many neoplasms, including prostate cancer [8–10]. Blocking IKK-mediated IĸBα degradation and NF-ĸB activation, repression of NF-ĸB transactivation potential and stabilization of IĸB has been shown to inhibit aberrant gene expression, malignant phenotypes and therapeutic resistance in pre-clinical models of prostate cancer [11–14]. Previous studies suggested that IKKβ subunit is essential for canonical NF-ĸB activation through mediation of IĸB degradation, leading to rapid development of IKKβ inhibitors [13, 14]. Bortezomib, a proteasome inhibitor that blocks IĸB degradation and RelA activation, and BS345541, an inhibitor of IKKβ kinase activity have been evaluated in the management of prostate cancer [15, 16]. Clinical trials using bortezomib alone in advanced stage prostate cancer and in combination with androgen blockade and chemotherapy showed limited clinical efficacy due to incomplete targeting of NF-ĸB/Rel subunits and other signaling pathways mediated through androgen receptor and β-catenin, which contribute to the resistance to bortezomib and castration in prostate cancer [17, 18]. Another recent study showed proteasome inhibition by bortezomib increases IL-8 expression and nuclear accumulation of IKKα [19]. Together, these findings suggest that drugs targeting IKKβ-mediated activation of NF-ĸB alone are insufficient. It has been hypothesized that IKKα may contribute to canonical and/or alternative NF-ĸB/Rel activation and promotion of malignant phenotype. The few previous studies on IKKα in prostate cancer have emphasized its potential role in controlling invasiveness and metastasis in IKK^AA/AA^/TRAMP mice [20, 21]. These studies emphasize that IKKα alone is sufficient to contribute to activation of NF-ĸB pathways, promoting a malignant phenotype and suggest that these mechanisms may be important therapeutic target in treating prostate cancer. In recent years, there has been considerable study of the functions of plant flavonoids in inhibition of the NF-ĸB pathway [22–25]. A better understanding of the target specificity and dosage required for inhibition of NF-ĸB signaling through IKK inhibitors may lead to the development of more specific and efficacious inhibitors of NF-ĸB signaling.

Apigenin (4′, 5, 7-trihydroxyflavone) is an important component of fruit and vegetable-rich human diets [26, 27]. Apigenin inhibits proliferation in several types of cancer cells and induces apoptosis [28–30]. Apigenin has been shown to suppress cytokine-induced NF-ĸB activation and cancer progression by blocking IKKβ activity [31]. Apigenin has also been shown to inactivate NF-ĸB through suppression of p65/RelA phosphorylation at Ser536 [32]. We have previously shown that apigenin suppresses constitutive and TNFα-induced NF-ĸB activation in human prostate cancer cells, which in turn block the downstream signaling related to cancer progression [33]. Apigenin intake by transgenic adenocarcinoma of the mouse prostate (TRAMP) mice inhibits prostate carcinogenesis and completely blocks tumor metastasis [34]. Consistent with the identification of IKKα as a therapeutic target and its involvement in prostate cancer, we hypothesized that suppression of IKKα kinase activation affecting downstream NF-ĸB signaling by apigenin might markedly reduce cancer progression. Therefore, we studied the molecular basis of the effect of apigenin on IKKα and IKKβ inhibition using human prostate cancer cell lines and in an athymic nude mouse xenograft model.

## RESULTS

### Aberrant expression and phosphorylation of IKKα and IKKβ in human prostate cancer specimens and cell lines

**Figure 1:**
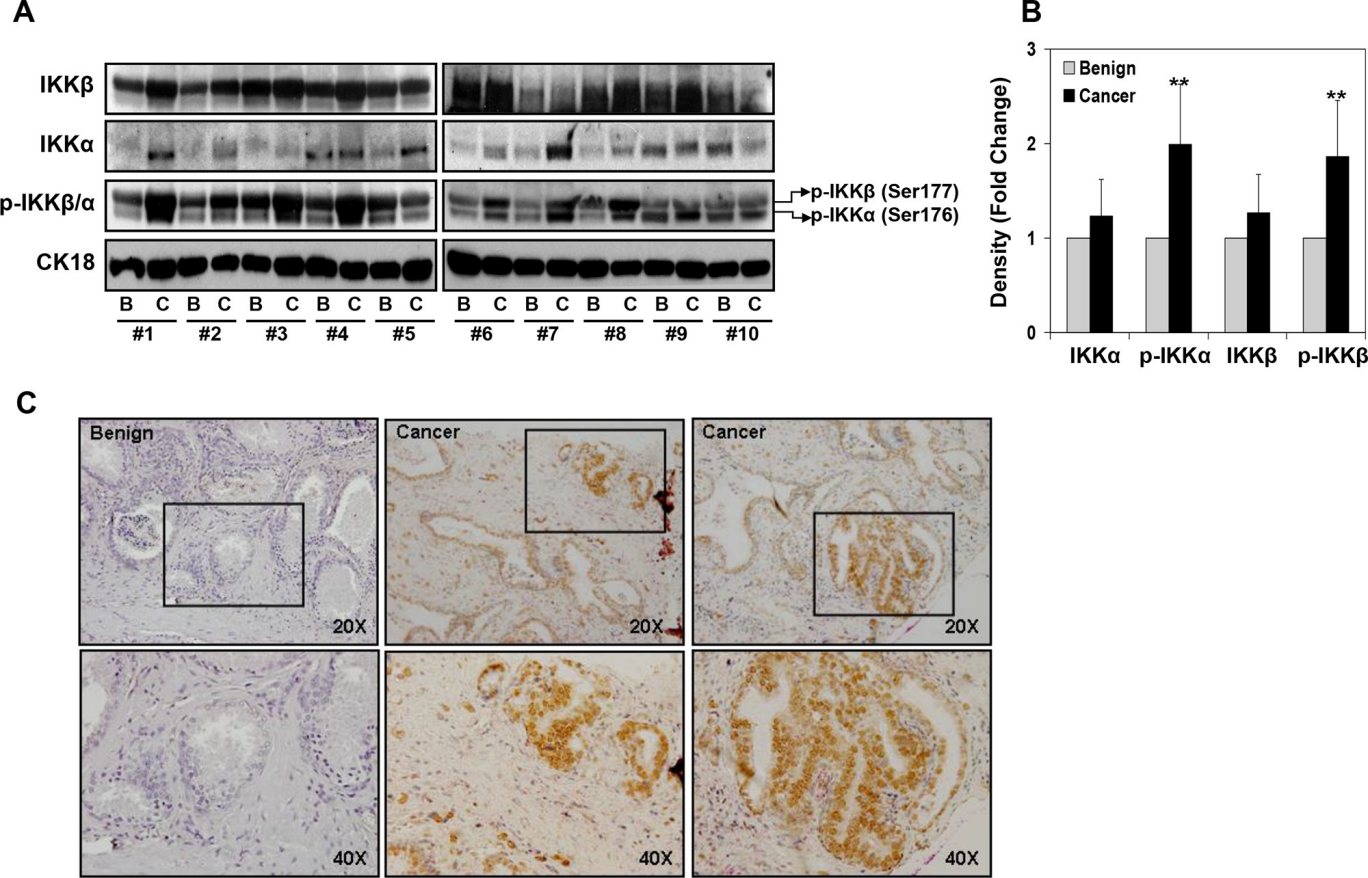
Expression of IKKα/β and their phosphorylation in various representative benign and prostate cancer tissues **A.** Protein expression of IKKα, IKKβ, *p*-IKKα (Ser176) and *p*-IKKβ (Ser177) in paired benign and cancer specimens was analyzed by Western blotting; cytokeratin18 expression served as loading control. A modest increase in IKKα and IKKβ expression was observed in cancer specimens compared to benign tissue; whereas a significant increase in *p*-IKKα and *p*-IKKβ was observed in cancer specimens. **B.** Relative density of bands showing protein expression in benign and cancer specimens. Mean ± SD; **P < 0.05, compared to benign tissue. **C.** Paraffin-embedded (4.0 μm) sections from benign and prostate cancer were used for *p*-IKKα/β (Ser177/176) expression by immunohistochemistry. Phosphorylated levels of IKKα/β was detected both in the nucleus and in the cytoplasm of malignant cells and was more intense in the cytoplasm. Magnified at x20 and x40. Details are described in ‘materials and methods’ section.

IKKα and IKKβ have 52% amino acid identity with a similar structural organization, which includes kinases, leucine zipper, and helix-loop-helix domains (Supplemental Figure 1). The kinase domains of those proteins contain MAP kinase activation loop with closely spaced serine residues at position 176 and 180 in IKKα and positions 177 and 181 in IKKβ. Phosphorylation of these serine residues by upstream kinases viz. MEKK1 and NIK activates IKK kinase activity [8–10]. To extend these observations we determined IKKα and IKKβ expression, their phosphorylation and localization in 10 clinical prostate cancer specimens (Gleason score 3 + 3 and 3 + 4) and matched benign tissue from same patients by Western blot analysis. As shown in Figure 1A, the total protein levels of IKKα and IKKβ were detected both in prostate cancer and benign specimens. A modest increase in IKKα and IKKβ expression was observed in cancer specimens compared to benign tissue. The phosphorylated levels of IKKα/β as *p*-IKKβ (Ser177) as upper band and *p*-IKKα (Ser176) as lower band was observed in these specimens. Densitometric analysis demonstrated 1.99-fold increase in *p*-IKKα and 1.86-fold increase in *p*-IKKβ levels in cancer tissue compared to benign tissue (Figure 1B).

Next we examined *p*-IKKα/β expression by immunochemical staining of paraffin-embedded sections of 24 prostate cancer specimens consisting of 14 low-grade tumor (Gleason score 5–6), 10 median-grade tumor (Gleason score 7–8) and 6 benign tissues (Figure 1C). The staining intensity and localization of *p*-IKK in tumor tissue was assessed by light microscopy. Over 90% of *p*-IKKα/β was detected both in the nucleus and in the cytoplasm of malignant cells and was more intense in the cytoplasm. Furthermore, phosphorylated form of IKKα/β was distributed both in the nuclear and cytosolic fractions of 4 human prostate cancer cells, where 22Rv1 and PC-3 cells exhibited high *p*-IKKα/β expression in the nuclear fraction as well as in the cytosol, compared to LNCaP and DU145 cells (Supplemental Figure 2). These findings suggest that IKK kinases are constitutively active in prostate cancer cells and clinical prostate cancer specimens, compared to benign tissue.

### Knockdown of IKKα and IKKβ cause cell cycle arrest and decrease proliferation in prostate cancer cells

**Figure 2:**
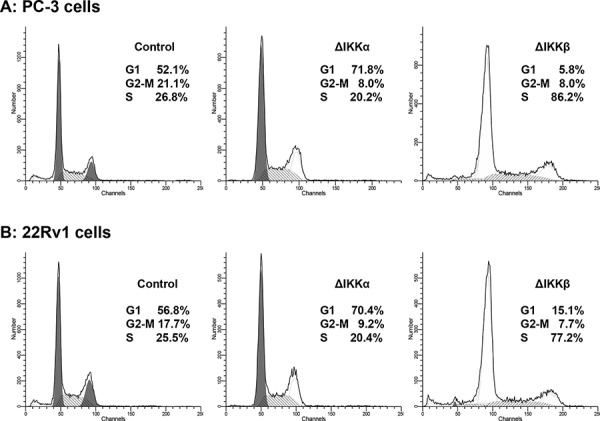
Silencing effect of IKKα and IKKβ on cell cycle in human prostate cancer cells **A.** PC-3 and **B.** 22Rv1 cells were transfected with IKKα and IKKβ shRNA retroviral particles, a pool of viral particle containing 3 target specific constructs and one scrambled and one with negative shRNA that encode 19–25 nt (plus hairpin) designed to knockdown gene expression, selected under polybrene and used after 15–20 passage, stained with propidium iodide (50 mg/ml) and subjected to cell cycle analysis by flow cytometry. Percentage of cells in G0-G1, S and G2-M phase were calculated using Mod-fit computer software and are represented in the right side of the histograms. Knockdown of IKKα in both cell lines resulted in significant accumulation of cells in the G1 phase whereas IKKβ knockdown resulted in arrest in S phase of the cell cycle. Details are described in ‘materials and methods’ section.

To determine the relevance of endogenous IKKα/β activation in cancer cell proliferation, we knockdown IKKα and IKKβ in human prostate cancer PC-3 and 22Rv1 cells using short hairpin RNA (shRNA) approach. IKKα and IKKβ expression was significantly downregulated by the use of IKKα and IKKβ shRNA2, whereas no significant change in IKKα and IKKβ was observed by scrambled shRNA or use of transfection reagent alone in both cell lines (Supplemental Figure 3). Knockdown of IKKα in PC-3 cells resulted in significant accumulation of cells in the G1 phase (71.8%) compared to 52.1% in control cells; and 70.4% in 22Rv1 cells, compared to 56.8% in control cells. Similarly, knockdown of IKKβ in both cell lines resulted in S-phase arrest of the cell cycle; PC-3 cells (86.2% versus 26.8%) and 22Rv1 cells (77.2% versus 25.5%) compared to their corresponding controls (Figure 2A & 2B). Together these results provide evidence that IKK are oncogenic and regulate proliferation in prostate cancer cells.

### Apigenin blocks catalytic site of IKKα and IKKβ-*In silico* molecular modeling

**Figure 3:**
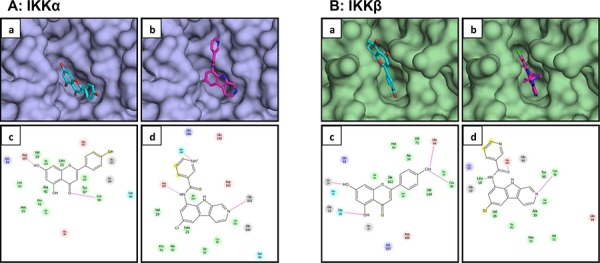
Molecular modeling of the interaction between apigenin and IKKα/β **A.** Apigenin **a.** and PS1145 **b.** docked in to the pocket of IKKα. Apigenin is represented as sticks with carbon atoms in cyan and oxygen atoms in red; PS1145 is represented in sticks with carbon atoms in magenta, nitrogen atoms in blue, and chlorine atom in green. Structure of IKKα is depicted as surface model. Schematic illustration of interaction between apigenin **c.** and PS1145 **d.** with different amino acid residues in the pocket of IKKα is demonstrated. **B.** Apigenin **a.** and PS1145 **b.** docked in to the pocket of IKKβ. Apigenin is represented as sticks with carbon atoms in cyan and oxygen atoms in red; PS1145 is represented in sticks with carbon atoms in magenta, nitrogen atoms in blue, and chlorine atom in green. Structure of IKKβ is depicted as surface model. Schematic illustration of interaction between apigenin **c.** and PS1145 **d.** with different amino acid residues in the pocket of IKKβ is shown. Details are described in ‘materials and methods’ section.

Our previous studies demonstrate that apigenin suppresses constitutive and TNFα-induced NF-ĸB activation in human prostate cancer cells [33]. Therefore we hypothesized that apigenin might regulate NF-ĸB activation by blocking IKK activity. We performed *in silico* docking studies with apigenin and PS1145, an IKK inhibitor to determine their effectiveness in suppressing kinase activity. Docking results show that both apigenin and PS1145 were docked to the deep cleft in the structure of IKKα (Figure 3A). Docked conformation of apigenin exhibit two fused aromatic rings toward the base of the pocket while the other aromatic ring protruding outwards (Figure 3A-a). Inside the pocket, apigenin is anchored by two hydrogen bonds – one between side chain of Asp165 and one of the hydroxyl groups in the buried phenyl ring; second between carbonyl oxygen in apigenin and backbone of Cys98. Two dimensional representation of interaction of apigenin with different amino acid residues in the pockets is shown in (Figure 3A-c). PS1145 was docked in the pocket of IKKα in similar mode as of apigenin. The docked conformation of PS1145 showed chlorine substituted ring buried deep inside the pocket of IKKα (Figure 3A-b). As in case of apigenin, hydrogen bond interaction of the ligand with carboxylic side chain of Asp165 has been observed. In addition, two other hydrogen bonds between ligand and protein stabilize the interaction between the two molecules. Nitrogen atoms in the two 6-membered rings form hydrogen bonds with delta oxygen of Asn149 and backbone of Gly101 (Figure 3A-d).

Next we performed *in silico* docking with IKKβ. Docking results show that both apigenin and PS1145 were docked to the deep cleft in the structure of IKKβ. Figure 3B show docked conformation of both the ligands into the pocket of IKKβ. It has been observed that apigenin is well anchored by hydrogen bonds with amino acid resides in the protein from both ends. All three hydroxyl groups in apigenin have been observed to be favorably oriented around different hydrogen bond acceptor atoms in the protein (Figure 3B-a). Two hydroxyl groups in one of the phenyl ring participate in hydrogen bonding with main chain atoms of Thr20 and Gly24. The single hydroxyl group in another phenyl ring interacts with backbone of Glu94 via hydrogen bond (Figure 3B-c). In case of PS1145 docking to IKKβ, three hydrogen bonded interaction have been observed between ligand and amino acid residues in the pocket of the protein (Figure 3B-b). Hydrogen bond is formed between carbonyl oxygen in ligand with main chain oxygen of Asp100. Further, backbone of Leu18 and Cys98 forms hydrogen bonds with two different nitrogen atoms of the ligand (Figure 3B-d). Overall, the program predicted superior binding ability of apigenin with IKK kinases, compared to PS1145. These predicted results were validated with the experimental data.

### Apigenin inhibits IKKα and IKKβ phosphorylation in prostate cancer cells

**Figure 4:**
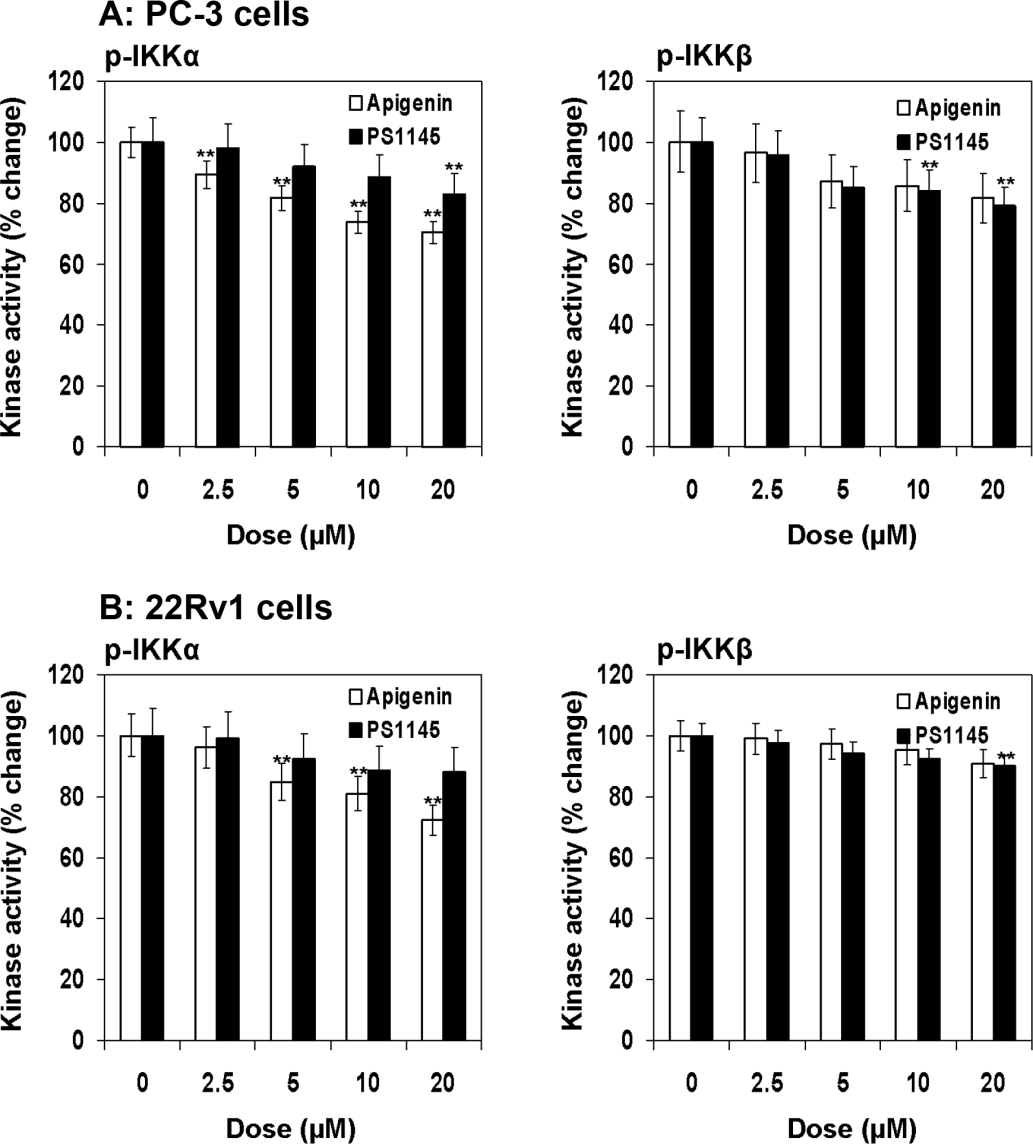
Effect of apigenin and PS1145 on IKKα and IKKβ phosphorylation in human prostate cancer cells **A.** PC-3 and **B.** 22Rv1 cells were treated with indicated doses with apigenin and PS1145 for 16 h and IKKα and IKKβ kinase activity was determined using PathScan^®^ Phospho-IKKα (Ser176/180) and PathScan^®^ Phospho-IKKβ (Ser177/181) Sandwich ELISA Kit following vendor's protocol. Kinase activity is depicted as fold change. A significant decrease in IKKα/β phosphorylation in dose-dependent fashion, which was more pronounced for IKKα than IKKβ. Mean ± SD; **P < 0.05, compared to vehicle treated control. Details are described in ‘materials and methods’ section.

Next we determined the effect of apigenin and PS1145 on phosphorylation of IKKα (Ser176/180) and IKKβ (Ser177/181) in prostate cancer PC-3 and 22Rv1 cells using Pathscan^®^ ELISA assay. As shown in Figure 4A & 4B, a dose dependent decrease in IKKα phosphorylation by apigenin which was more pronounced than PS1145 in both cell lines. Treatment with 2.5 to 20 μM apigenin concentration in PC-3 cells resulted in 89.2% to 70.3% and in 22Rv1 cells 96.2% to 72.4% decrease in IKKα phosphorylation. Similarly, PS1145 treatment at similar doses in PC-3 cells resulted in 98.0% to 83% and in 22Rv1 cells 97.1% to 88.3% decrease in IKK phosphorylation. The effect of apigenin in the inhibition of IKKβ phosphorylation was similar to that of PS1145 in both cell lines. Treatment with 2.5 to 20 μM apigenin concentration in PC-3 cells resulted in 96.4% to 81.6% and in 22Rv1 cells 99% to 91% decrease in IKKβ phosphorylation; whereas PS1145 treatment of PC-3 cells resulted in 95.9% to 78.9% and 98% to 90% in 22Rv1 cells at similar doses ranging from 2.5 to 20 μM. Taken together, these results suggest that IKKα is preferential target of apigenin which has higher potential to inhibit IKKα phosphorylation than IKKβ.

### Apigenin binds to IKKα with higher affinity than IKKβ-*Ex vivo* study

**Figure 5:**
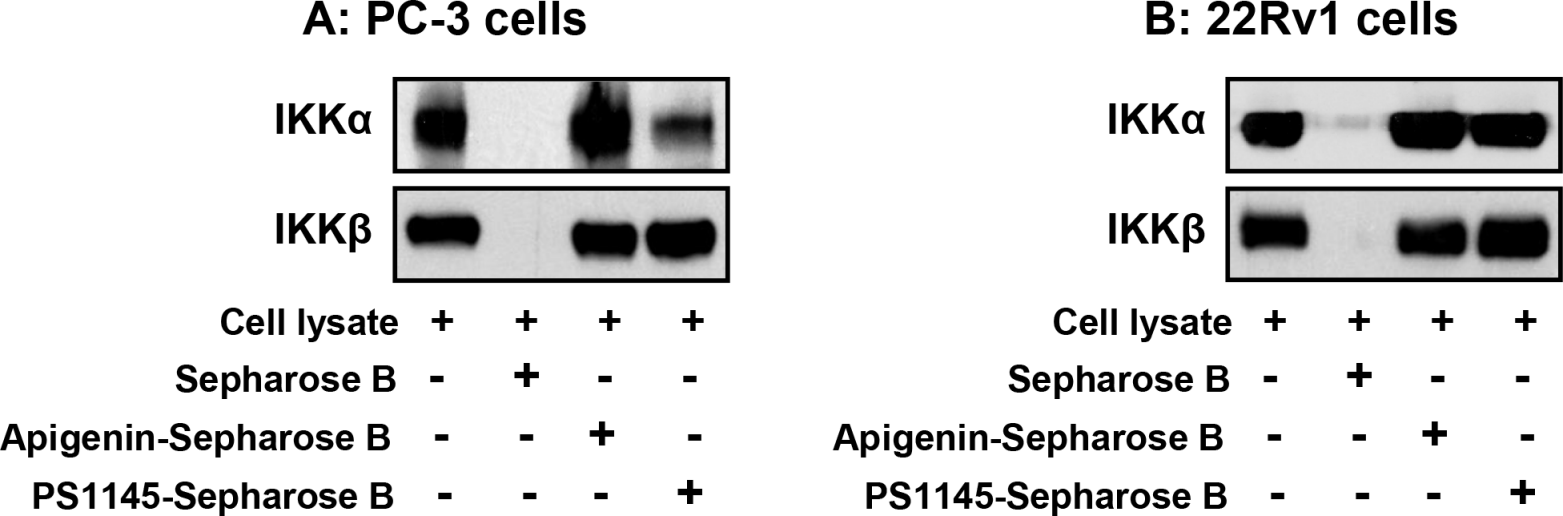
Apigenin binding to IKKα and IKKβ by *ex vivo* pull down assay **A.** PC-3 and **B.** 22Rv1 cells were used for whole cell lysate precipitated with sepharose 4B beads, sepharose 4B-apigenin and sepharose 4B-PS1145 coupled beads. Whole cell lysate (input control, lane 1), precipitate with sepharose 4B beads (negative control, lane 2), sepharose 4B-apigenin coupled beads (lane 3) and sepharose 4B-PS1145 coupled beads were applied to SDS-PAGE, and detected with antibodies against IKKα and IKKβ after transferring the membrane. An increased binding of IKKα was observed in sepharose B-apigenin coupled beads in both cell lines whereas modest binding was observed for IKKβ with apigenin. Details are described in ‘materials and methods’ section.

Next we determined whether the inhibition of IKKα and IKKβ phosphorylation by apigenin was due to direct interaction, we investigated binding of apigenin to IKKα and IKKβ in *ex vivo* approach using sepharose B beads (Figure 5A & 5B). As a positive control, IKKα and IKKβ was detected in high levels in the cell lysates obtained from PC-3 and 22Rv1 cells (Lane 1) but was not detected in sepharose B beads alone (Lane 2). An increased binding of IKKα was observed in sepharose B-apigenin coupled beads (Lane 3) in both PC-3 and 22Rv1 cells whereas modest binding was observed for IKKβ with apigenin. Experiment performed with PS1145 demonstrate increased binding of IKKβ with sepharose B-PS1145-coupled beads, whereas no significant affinity binding was noted for IKKα with PS1145 in both cell lines (Lane 4). These data demonstrate that apigenin interacts with cellular IKKα with higher affinity than IKKβ.

### Apigenin inhibits IKKα/β phosphorylation and suppresses NF-ĸB activation in prostate cancer cells

**Figure 6:**
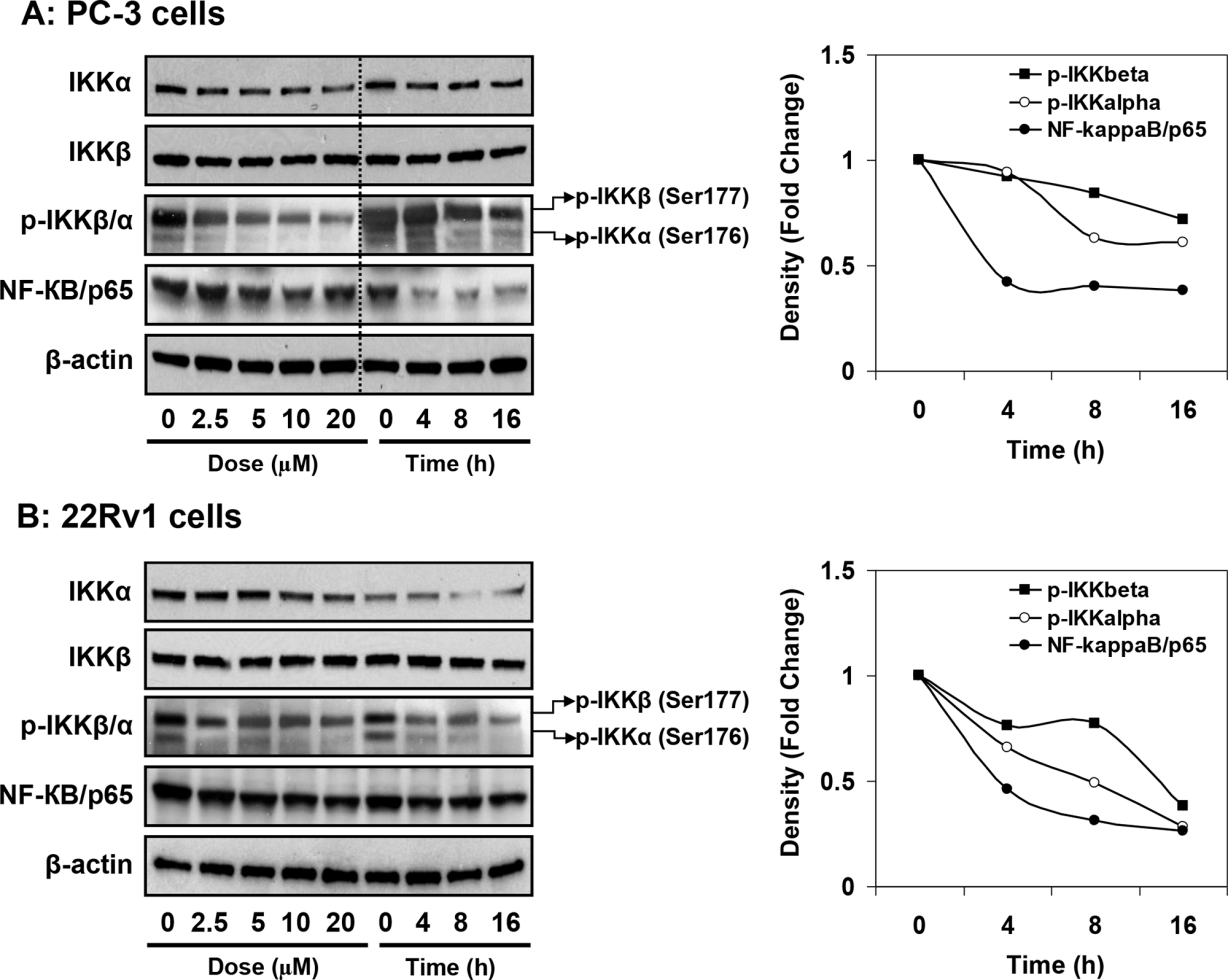
Effect of apigenin on IKKα/β its phosphorylation and NF-ĸB/p65 protein expression in human prostate cancer cells **A.** PC-3 and **B.** 22Rv1 cells were treated with indicated doses and times with 20 μM apigenin for 16 h and protein expression of NF-ĸB/p65, IKKα, IKKβ and their phosphorylation was determined by Western blot analysis. A significant decrease in IKKα/β phosphorylation, IKKα and NF-ĸB/p65 in dose- and time- dependent fashion was observed. Relative density of bands showing time course change in the protein expression of *p*-IKKα/β and NF-ĸB/p65 is shown in the right panel. Details are described in ‘materials and methods’ section.

Next we determined the inhibitory effect of apigenin on IKKα and IKKβ and their phosphorylation. As shown in Figure 6A & 6B, treatment of PC-3 and 22Rv1 cells with 2.5, 5, 10 and 20 μM doses of apigenin for 16 h resulted in significant decrease in IKKα and *p*-IKKα/β in dose-dependent fashion, which was more pronounced for *p*-IKKα than p-IKKβ. Similar effects were observed upon treatment of cells with 20 μM apigenin in time-dependent fashion where decrease in IKKα/β phosphorylation was observed at 4, 8 and 16 h, respectively. No significant change in the levels of IKKβ was observed in these cell lines. Furthermore, apigenin treatment resulted in marked inhibition of NF-ĸB/p65 protein expression in dose- and time- dependent manner in both cell lines.

**Figure 7:**
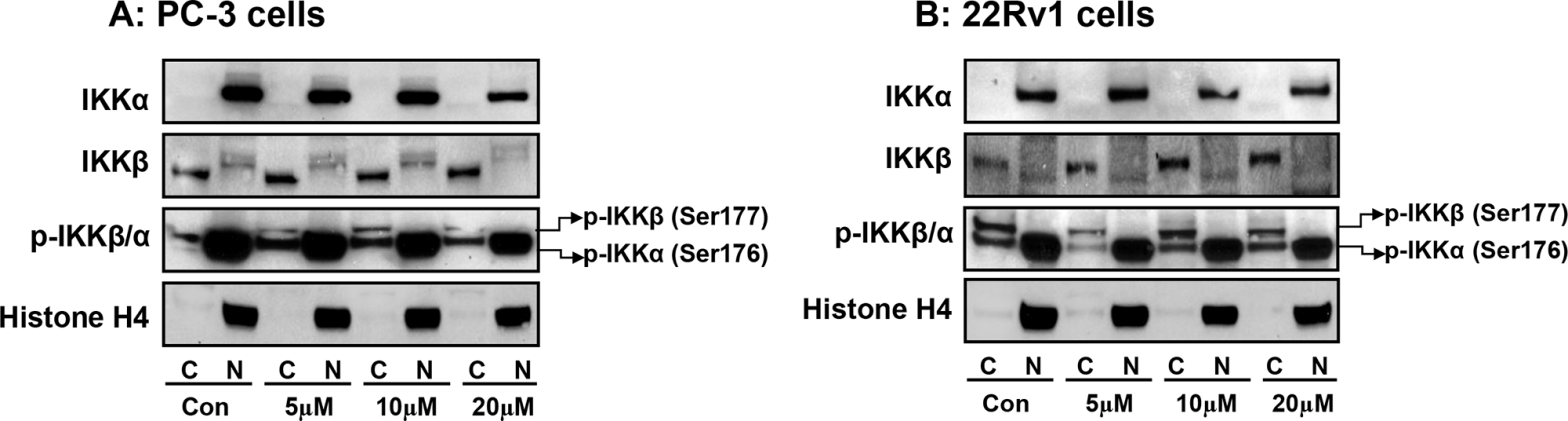
Effect of apigenin on sub-cellular distribution of IKKα, IKKβ and its phosphorylated forms in human prostate cancer cells **A.** PC-3 and **B.** 22Rv1 cells were treated with indicated doses of apigenin for 16 h; subjected to preparation of cytosolic and nuclear fractions and protein expression of IKKα, IKKβ and their phosphorylation was determined by Western blot analysis. A significant decrease in *p*-IKKα in the nuclear fraction and simultaneous increase in *p*-IKKα expression in the cytosol after apigenin treatment, compared to untreated group in both cell lines. Details are described in ‘materials and methods’ section.

We also determined the effect of apigenin on cytosolic and nuclear changes in IKKα/β phosphorylation in PC-3 and 22Rv1 cells. As shown in Figure 7A & 7B, apigenin treatment significantly suppressed phosphorylation of IKKα in the nuclear fraction, compared to untreated group in both cell lines. These events resulted in increased *p*-IKKα expression in the cytosol after apigenin treatment. No significant effect was observed on the protein levels of IKKα and IKKβ and *p*-IKKβ after apigenin treatment in the nucleus and the cytosol.

### Apigenin causes cell cycle arrest in prostate cancer cells

**Figure 8:**
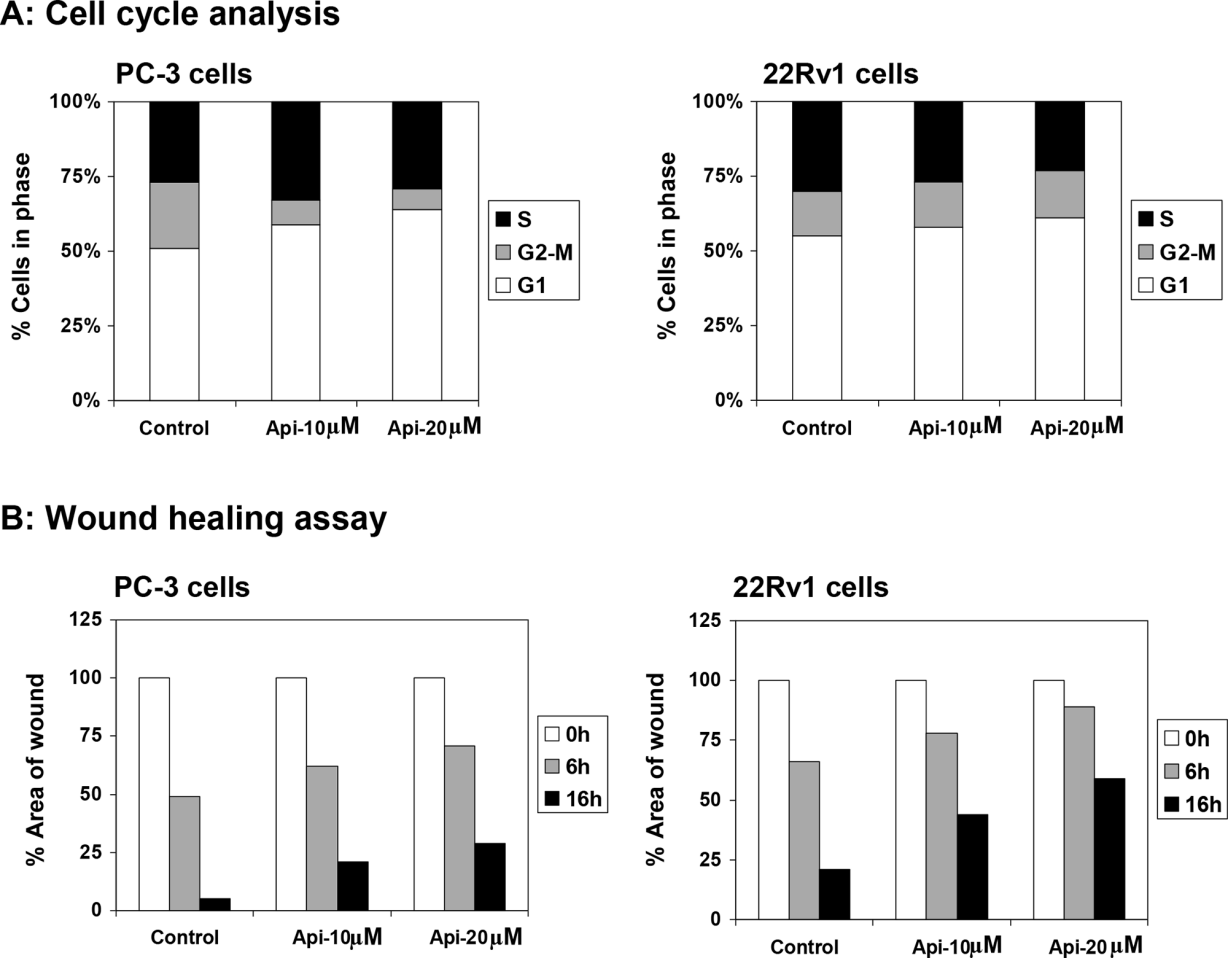
Effect of apigenin on DNA cell cycle and wound healing in prostate cancer cells **A.** DNA cell cycle analysis. PC-3 and 22Rv1 cells were synchronized in G0 phase by depleting the nutrients for 36 h (referred as control) and replating at sub confluent densities into complete medium containing vehicle or apigenin at indicated doses for 16 h, stained with PI (50 mg/ml) and analyzed by flow cytometry. Percentage of cells in G0-G1, S and G2-M phase were calculated using Mod-fit computer software and are represented in the right side of the histograms. A marked increase in G0-G1 phase accumulation of cells was observed after apigenin treatment. **B.** Wound healing assay. PC-3 and 22Rv1 cells were seeded into six-well plates and grown overnight. Then the cells were serum starved for 24 h. A sterile 200 μl pipette tip was used to scratch the cells to form a wound. The cells were washed with PBS and treated with vehicle or apigenin at indicated doses for 6 h and 16 h. Migration of the cells to the wound was visualized with an inverted Olympus phase-contrast microscope. A decrease in wound healing was observed after apigenin treatment in both cell lines. Details are described in ‘materials and methods’ section.

Next we determined whether apigenin-mediated decrease in IKKα phosphorylation causes perturbation in cell cycle and proliferation in cancer cells. We ascertained the effect of apigenin on the cell cycle. The cells were synchronized by serum deprivation for 36 h and later incubated with 10% fetal bovine serum with varying concentrations of apigenin for 16 h. Compared with the untreated controls, apigenin treatment resulted in an appreciable arrest of PC-3 cells in G_0_-G_1_ phase of cell cycle after 16 h of the treatment. The treatment caused an arrest of 59% cells in G_0_-G_1_ phase of the cell cycle at 10 μM concentration that further increased to 65% at 20 μM in these cells, compared with vehicle-treated control (51%). This increase in G_0_-G_1_ cell population was accompanied with a concomitant decrease of cell number in S phase and G_2_-M phase of the cell cycle. Similarly, apigenin treatment to 22Rv1 cells caused an arrest of 58% cells in G_0_-G_1_ phase of the cell cycle at 10 μM concentration that further increased to 61% at 20 μM in these cells, compared with vehicle-treated control (55%) (Figure 8A). These results suggest that apigenin perturbs cell cycle progression of prostate cancer cells.

### Apigenin suppresses migration in prostate cancer cells

Next, we determined the effects of apigenin on migration of human prostate cancer PC-3 and 22Rv1 cells by means of wound-healing assay. As shown in Figure 8B, wound-healing assay demonstrated that apigenin diminished migration of human prostate cancer cells. Treatment of PC-3 cells with 10 μM and 20 μM apigenin for 6 h resulted in 62% and 71% of open wound area, compared to untreated cells (49%); whereas 16 h apigenin treatment at similar doses resulted in 21% and 29% open wound area, compared to 5% in untreated cells. Similarly in 22Rv1 cells, 78% and 89% of open wound area was observed in 10 μM and 20 μM apigenin, compared to 66% in untreated cells at 6 h; whereas apigenin treatment at similar doses resulted in 44% and 59% open wound area, compared to 21% in untreated cells after scratching.

### Apigenin suppresses tumor growth in athymic nude mouse xenograft model

Apigenin has been shown to be effective in cell culture, inhibiting IKKα/β phosphorylation and downstream NF-ĸB signaling in human prostate cancer PC-3 and 22Rv1 cells; therefore, we extended our study to determine whether these events occur *in vivo* using xenograft mouse model. We designed a protocol that simulates a therapy regimen, wherein apigenin was provided at 20 and 50 μg/mouse/day through gavage, initiating 2 weeks after cell inoculation and continuing for 8 weeks. In this experimental protocol, intake of apigenin inhibited the growth of tumor xenograft at both doses of apigenin. As shown in Supplemental Figure 4A, PC-3 tumor volume was inhibited by 32% and 51% (*P* < 0.005 and 0.0001) and the wet weight of tumor was decreased by 28% and 40% (*P* < 0.001) after 20 and 50 μg/day apigenin, respectively, at the termination of the experiment. Similarly, apigenin intake resulted in 40% and 53% (*P* < 0.05 and 0.002) decrease in 22Rv1 tumor volume and the wet weight was decreased by 29% and 42% (*P* < 0.001) (Supplemental Figure 4B). Apigenin treatment also resulted in significant enhancement of apoptosis as measured by M30 reactivity in tumor lysates from PC-3 and 22Rv1 (Supplemental Figure 5A & 5B). Furthermore, apigenin intake by these mice did not seem to induce any adverse effects as judged by monitoring body weight gain, dietary intake and prostate weight (data not shown).

### Apigenin intake causes decrease tumor proliferation and increase apoptosis through inhibition of IKK phosphorylation

**Figure 9:**
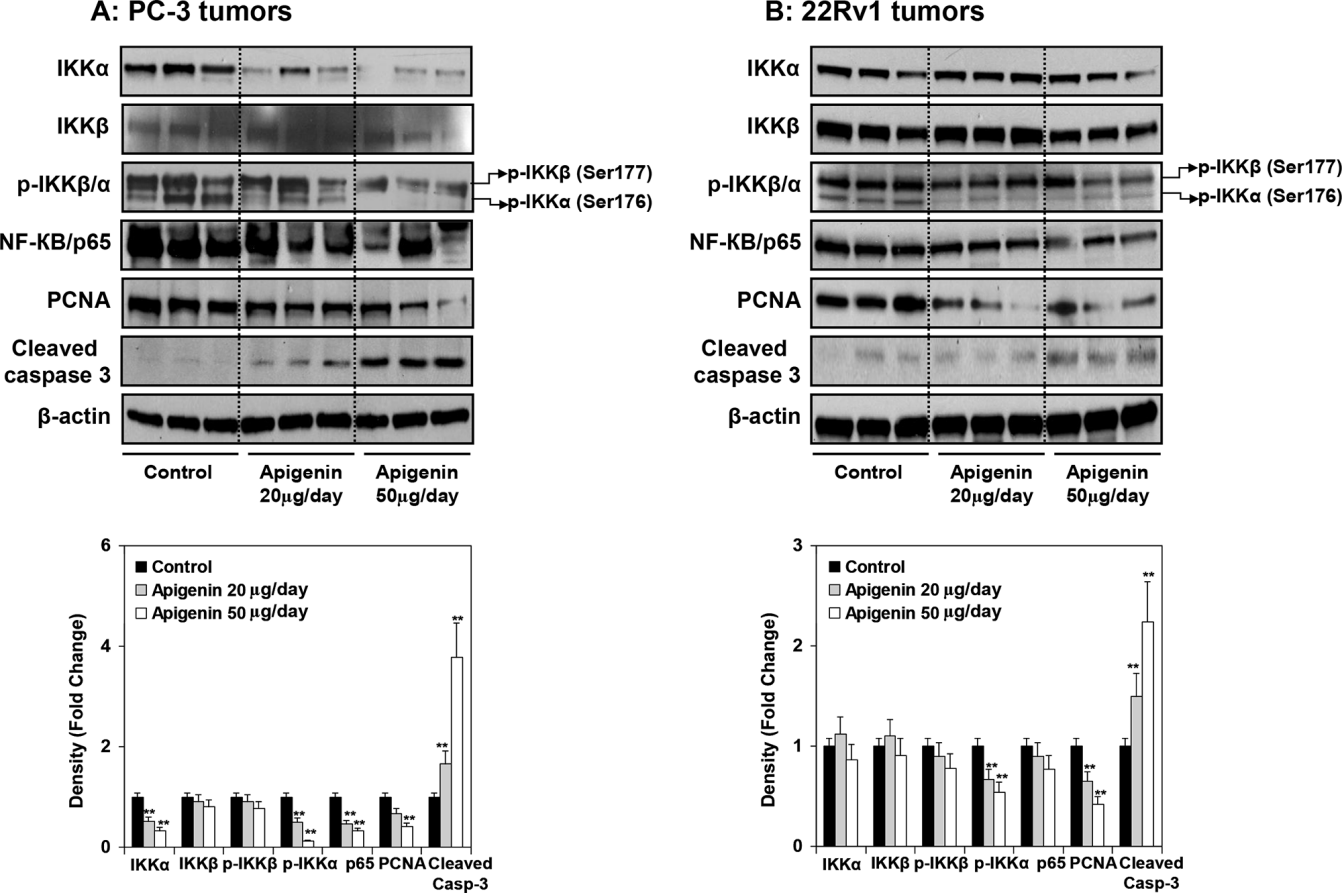
Effect of apigenin intake on the protein expression of IKKα/β and its phosphorylation, NF-ĸB/p65, and markers of proliferation and apoptosis in prostate tumor xenograft specimens obtained from athymic nude mice **A.** PC-3 and **B.** 22Rv1 tumors obtained after tumor implantation and feeding mice with 20- and 50- μg apigenin in 0.2 ml vehicle daily for 8 weeks. Details are described in Supplemental figure 4. Vehicle treated group served as control. Protein expression of IKKα, IKKβ, *p*-IKKα/β, NF-ĸB/p65, proliferating cell nuclear antigen (PCNA) and cleaved caspase 3 were determined by Western blot analysis. A marked reduction in the protein expression of IKKα and its phosphorylation, NF-ĸB/p65 whereas a modest decrease in *p*-IKKβ in PC-3 and 22Rv1 tumor xenografts was observed after apigenin intake. A dose-dependent decrease in proliferating nuclear cell antigen (PCNA), and increase in the expression of cleaved caspase 3 was observed in both tumor xenografts. Relative density of bands showing fold change in the protein expression of these protein is shown below. Mean ± SD; **P < 0.05, compared to vehicle treated control. Details are described in ‘materials and methods’ section.

**Figure 10:**
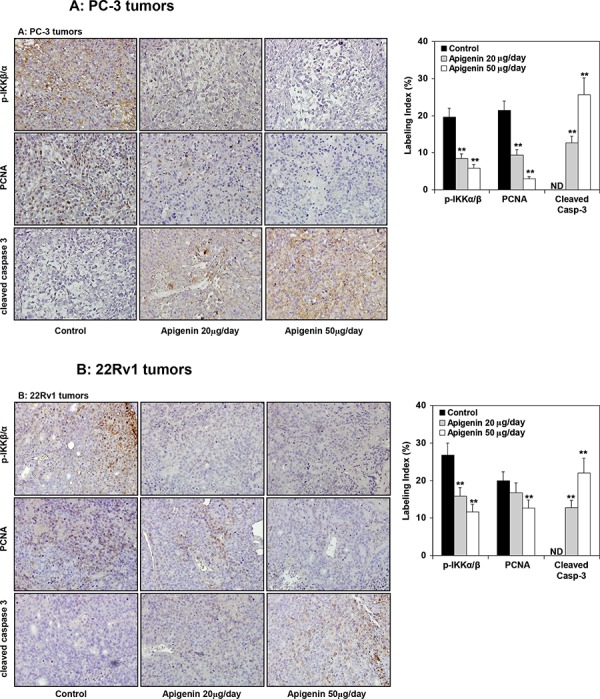
Effect of apigenin intake on the IKKα/β phosphorylation and extent of proliferation and apoptosis in prostate tumor xenograft specimens obtained from athymic nude mice **A.** PC-3 tumors and **B.** 22Rv1 tumors. Immunohistochemical analyses of *p*-IKKα/β, PCNA and cleaved caspase 3 was performed in mice fed with 20- and 50- μg apigenin in 0.2 ml vehicle daily for 8 weeks. Details are described in Supplemental figure 4. Vehicle treated group served as control. A significant decrease in IKKα/β phosphorylation, marked decrease in proliferation index and enhancement of apoptosis was observed after apigenin intake in PC-3 and 22Rv1 tumor xenografts, compared with control group. Labeling index for *p*-IKKα/β, proliferation and apoptotic index is shown in the panel on the right. Mean ± SD; **P < 0.05, compared to vehicle treated control. Details are described in ‘materials and methods’ section.

At the termination of the study, xenografts were examined for expression of IKK and its phosphorylation, NF-ĸB/p65 and the extent of tumor proliferation and apoptosis. As shown in Figure 9A & 9B, oral intake of apigenin at doses of 20 and 50 μg resulted in marked reduction in the protein expression of IKKα and its phosphorylation, NF-ĸB/p65 whereas a modest decrease in *p*-IKKβ in PC-3 and 22Rv1 tumor xenografts. A dose-dependent decrease in proliferating nuclear cell antigen (PCNA), a marker of proliferation and increase in the expression of cleaved caspase 3, an apoptosis marker was observed in both tumor xenografts. As shown in Figure 10A, compared with controls, PC-3 xenograft samples from apigenin-fed groups showed a marked decrease in IKKα/β phosphorylation and PCNA staining. The quantification of *p*-IKKα/β and PCNA-positive cells in PC-3 tumor sections showed that oral intake of apigenin at both doses (20 and 50 μg) results in 57% and 70% (*P* < 0.001) decrease in IKKα/β phosphorylation and 56% (*P* < 0.001) and 86% (*P* < 0.001) decrease in proliferation index compared with control group. In case of cleaved caspase 3, tumor xenografts from apigenin-fed groups showed a marked increase in cleaved caspase-3 positive cells compared with control group. The quantification of cleaved caspase 3 stained samples showed that there was 13% (*P* < 0.001) and 26% increase (*P* < 0.001) in the number of positive cells in tumor sections from animals fed with apigenin at the dose levels of 20 and 50 μg, respectively, over that of control group. Similar results were obtained in 22Rv1 tumors where apigenin intake resulted in 41% and 56% (*P* < 0.003) for *p*-IKKα/β; 16% and 37% (*P* < 0.005) decrease in positive stained cells; whereas 12% and 22% (*P* < 0.002) increase in cleaved caspase-3 stained cells in 22Rv1 tumor xenograft compared to the control group. Representative photographs for *p*-IKKα/β, PCNA and cleaved caspase 3 positive cells in control and apigenin groups are shown at × 400 magnification (Figure 10B).

## DISCUSSION

In this study, we present several lines of evidence that IKKα plays an important role in cancer progression and that apigenin specifically blocks IKKα activation, leading to decreased proliferation and enhanced apoptosis in prostate cancer cells. First we show that constitutive IKKα levels and its activity is higher in cancer specimens compared to benign counterparts. Second we demonstrate that stable knockdown of IKKα causes G0-G1 phase cell cycle arrest. Third, our data indicate that apigenin directly binds to IKKα, inhibiting its kinase activity. Finally, we demonstrate that apigenin suppresses IKK and NF-ĸB activation, thereby causing reduced proliferation and induction of apoptosis in cell culture and in an *in vivo* tumor xenograft model.

In recent years cancer researchers have become increasingly interested in developing IKK inhibitors that work on various modes in the NF-ĸB signaling pathway [35–37]. After it was shown that IKKβ is more critical than IKKα in activating the NF-ĸB pathway, a number of chemical entities that commonly function as IKKβ selective inhibitors were developed and tested for anti-cancer efficacy [38, 39]. Although these molecules were efficient in suppressing inflammatory diseases, their usefulness as anticancer agents was limited, and it became clear that they lacked activity against IKKα. Recent studies have elucidated the unique role of IKKα in the activation of the alternative NF-ĸB pathways and have demonstrated that IKKα is a driver of the metastatic process [20, 21]. These studies imply that IKKα might be an attractive target for anticancer therapy. Given the role for IKKα-mediated NF-ĸB signaling in tumorigenesis and the IKKα activation in prostate cancer we provide *in vitro* experimental evidence that apigenin specifically binds with IKKα with higher affinity than IKKβ and blocks IKKα activation, which correlated with reduced migration of cancer cells treated with apigenin. Further research work is needed to investigate how apigenin-mediated IKKα inhibition affects downstream mechanisms involved in the process of metastasis.

Although studies have shown that IKKα plays a major role in tumor cell invasiveness and metastasis, its role in oncogenesis is controversial. Some studies highlight IKKα as a tumor suppressor rather a tumor promoter gene. Kwak *et al*. have shown that cells lacking IKKα show nuclear cyclin D1 overexpression and a neoplastic phenotype [40]; whereas increase in IKKα expression suppresses tumor progression and improves prognosis in nasopharyngeal carcinoma [41]. On the contrary, studies by Karin and colleagues have demonstrated that IKKα regulates mammary epithelial proliferation [42], and mutation-preventing IKKα activation inhibits metastasis in IKK^AA/AA^/TRAMP mice, a transgenic mouse model of prostate cancer [21]. Furthermore, Ammirante *et al*. have shown the requirement of IKKα for androgen-dependent expansion of epithelial progenitors responsible for prostate regeneration as well as in tumor recurrence [43]. We performed cell cycle analysis after knockdown of IKKα by shRNA. Our studies demonstrate an arrest of prostate cancer cells in G0-G1 phase of the cell cycle, in comparison to a control group, which was similar to the effect elicited after exposure of cancer cells to apigenin. These studies highlight the importance of IKKα in oncogenesis and further suggest that the tumor promotion and tumor suppressor activity of IKKα might be cell type specific. However, further investigation is needed in this area of research.

IKKα/β are essential regulators of the NF-ĸB pathway [5–7]. Although IKKα is not significantly involved in the phosphorylation of IĸBα resulting in its ubiquitination and subsequent degradation by the proteasome, it contributes to NF-ĸB activation through an unknown mechanism. Studies have shown that IKKα can phosphorylate IKKβ, which may have enhancing effects on NF-ĸB activation [44]. Our data presented in clinical prostate specimens demonstrate that both *p*-IKKα and *p*-IKKβ levels were higher in cancer specimens, which may contribute to NF-ĸB activation during cancer progression. Furthermore, our studies with apigenin have shown preferential binding and its effect on IKKα activation is more pronounced than IKKβ. Thus this dual inhibition of IKKα/β by apigenin would appear to be an optimal approach to block NF-ĸB activity. More detailed studies are warranted to identify the related molecular mechanisms responsible for this effect. Accumulating evidence suggests that the cellular effects of apigenin may be mediated by their interactions with specific proteins central to intracellular signaling cascades [45]. Apigenin has been shown to interact with a number of proteins kinases to regulate multiple cell signaling pathways [46–48].

Although our studies focused on IKK as an important target for apigenin, there are some other possible mechanisms of apigenin effects on tumor growth inhibition. Studies from our group and others have shown that the effects of apigenin are mediated by different pathways, such as focal adhesion kinase/Src signaling, the PI3K/Akt pathway, β-catenin/c-myc, estrogen receptor and others [48–52]. We have demonstrated that apigenin preferentially accumulates in the nuclear matrix and binds to nucleic acid base, endorsing its antioxidant function [53]. Recent study show that apigenin binds to heterogeneous nuclear ribonucleoprotein A2 and then modulates the activity of a large number of downstream cellular genes [54]. Accumulated evidence leads us to hypothesize that there is some distinct mechanism by which apigenin suppresses prostate cancer growth, and we believe this warrants further investigation.

In summary, our findings present evidence that apigenin inhibits IKKα-mediated NF-ĸB activation in prostate cancer. Moreover, apigenin intake effectively reduces the growth of human prostate tumor xenograft in a nude mouse model. These results suggest that suppression of IKKα/β activation by apigenin may be a useful strategy in the prevention and/or treatment of prostate cancer.

## MATERIALS AND METHODS

### Cell lines and treatments

Human prostate cancer PC-3 and 22Rv1 cells obtained from American Type Culture Collection (Manassas, VA) were used in the study. These cell lines possess high constitutive IKK activity. The cells were maintained in RPMI 1640 containing 2.05 mM L-glutamine (Lonza Walkersville, MD) with 10% fetal bovine serum, respectively, supplemented with 1% penicillin and streptomycin in a humidified incubator at 37°C with an atmosphere of 5% CO_2_. For experimental studies, these cells were grown to 70% confluence in monolayer and treated with apigenin at concentration ranging from 2.5 to 20 μM obtained from Sigma-Aldrich, St. Louis, MO (Cat# A3145; >97% purity) for 4–16 h in dimethyl sulfoxide as vehicle. The final concentration of the vehicle dimethyl sulfoxide did not exceed 0.1% in all the treatments.

### Human prostate tissue specimens

Both benign and prostate cancer tissue and paraffin-embedded block sections of human prostate cancer were obtained from the Tissue Procurement Facility of University Hospitals Case Medical Center and the Midwestern Division of the Cooperative Human Tissue Network. No consent was obtained for these discarded tissues per their hospital policies and Institutional Review Board protocols. These studies were approved by the Institutional Review Board at the University Hospitals Case Medical Center. Patients from whom these tissues were procured had undergone surgical procedures for prostatic disease and had not received any form of adjuvant therapy. The Gleason grade and score of adenocarcinoma in tissue specimens were assigned by a surgical pathologist experienced in genitourinary pathology. Immediately after procurement, samples were snap frozen in liquid nitrogen and stored at −80°C in the vapor phase of liquid nitrogen until further use.

### Transfection and generation of stable cell lines

Human prostate cancer PC-3 and 22Rv1 cells were transduced by IKKα and IKKβ shRNA retroviral particles, which is a pool of viral particle containing 3 target specific constructs and one scrambled and one with negative shRNA that encode 19–25 nt (plus hairpin) designed to knockdown gene expression (OriGene Technologies, Inc., Rockville, MD) following vendor's protocol. Briefly, cells were plated in a 6 well plates 24 h prior to viral infection. Transductions were carried out in RPMI containing 10% FBS complete medium and cells were incubate 48 h supplemented with polybrene (4 μg/ml). After 48 h stably transduced population were selected in 0.5–1 μg/ml puromycin for 2 week. To maintain authenticity of the stable cell lines, the cells were always maintained in the presence of selection agent and the cells were used after 15–20 passage.

### Docking of ligands to IKKα and IKKβ proteins

Three dimensional structural models of IKK proteins were built by homology modeling using Modeller 9v8. Crystal structures of calcium-dependent protein kinase domain from *Toxoplasma gondii* (PDB ID 3IS5) and protein kinase A from *Bos taurus* (PDB ID 1Q61) were used as templates for modeling IKKα and IKKβ, respectively. These modeled structures were subsequently used for protein-ligand docking studies. A receptor energy grid was generated around the ATP-binding pocket of the two proteins using Glide (Schrodinger, LLC). Coordinates for apigenin were extracted from its co-crystal structure with a different protein (PDB ID 3CF9). For PS1145, coordinates were downloaded from PubChem database (ID#16219884). The geometry of these compounds were optimized by energy minimization using Maestro (Schrodinger, LLC). LigPrep (Schrodinger, LLC) was used to assign appropriate charges and add hydrogen atoms to the ligand. Both of ligands were individually docked into the pocket of IKKα and IKKβ using Glide (Schrodinger, LLC) in XP (extra precision) mode.

### Quantitative determination of IKKα and IKKβ activity by ELISA

IKKα and IKKβ kinase activity was determined using PathScan^®^ Phospho-IKKα (Ser176/180) Sandwich ELISA Kit (Cat #7073) and PathScan^®^ Phospho-IKKβ (Ser177/181) Sandwich ELISA Kit (Cat #7080) following vendor's protocol. Briefly, adherent cells approximately 70–80% confluent, were washed with ice-cold 1X PBS. Cells lysed by adding, lysis buffer containing 1 mM PMSF and incubated on ice for 5 min. Later cells were scraped off and sonicated on ice, and centrifuged for 10 min at 12,000 *g* at 4°C. The supernatant was collected and 50 μl of cell lysate was added to the appropriate well, incubated for 2 h at room temperature. Wells were washed 4 times with 1X wash buffer followed by incubation with antibody and detection reagent followed by at 425 nm on a spectrophotometer.

### *Ex vivo* pull-down assay

For pull-down assay, 3 mg of apigenin was coupled to CNBr-activated sepharose 4B beads (25 mg) in a coupling buffer [0.5 M NaCl and 35% DMSO (pH 8.3)] for overnight at 4°C. The mixture was washed in 5 volumes of coupling buffer and then centrifuged at 1000 rpm for 3 min at 4°C. Precipitate was resuspended in 5 volumes of 0.1 M Tris-HCl buffer (pH 8.0) with 2 h rotation at room temperature. After washing three times with 0.1 M acetate buffer (pH 4.0) containing 0.5 M NaCl, and finally mixture was washed with 0.5 M containing NaCl in 0.1 M Tris-HCl (pH 8.0) buffer. Lysates from PC-3 and 22Rv1 cells (500 μg) was incubated at 4°C overnight with sepharose 4B beads or sepharose 4B-apigenin coupled beads (100 ml, 50% slurry) in a reaction buffer [50 mM Tris (pH 7.5), 5 mM EDTA, 150 mM NaCl, 1 mM DTT, 0.01% Nonidet *P*-40, 2 μg/ml BSA, 0.02 mM PMSF, and 1 mg protease inhibitor cocktail]. The beads were then washed 5 times with 50 mM Tris (pH 7.5) buffer containing 5 mM EDTA, 200 mM NaCl, 1 mM DTT, 0.02% Nonidet *P*-40, and 0.02 mM PMSF. Whole cell lysate (input control), lysates with sepharose 4B beads alone (negative control) or with sepharose 4B-apigenin coupled beads were applied to SDS-PAGE, and detected with antibody against IKKα and IKKβ, respectively, after transferring to membrane.

### Western blot analysis

Excised tumor tissues from xenograft implant and cells from treated and control groups were subjected to preparation of total lysate and isolation of cytosolic and nuclear fractions as described previously [34, 46]. For Western blotting, 25 μg of protein was resolved over 4–20% Tris-glycine polyacrylamide gel and then transferred onto the nitrocellulose membrane. The blots were blocked using 5% non-fat dry milk and probed using appropriate primary antibodies overnight at 4°C. The membrane was then incubated with appropriate secondary antibody horseradish peroxidase conjugate (Santa Cruz Biotechnology, Santa Cruz, CA) followed by detection using chemiluminescence ECL kit (GE Healthcare Biosciences). For equal loading of proteins, the membrane was probed with appropriate loading controls. The antibodies used were anti-IKKα (Cat#2682), anti-IKKβ (Cat#2678), anti-*p*-IKKα/β (Cat#2697), anti-cleaved caspase-3 (Cat#9661) and anti-histone H4 (Cat#2592) from Cell Signaling Technology, Danvers, MA. Anti-NF-ĸB/p65 (sc-8008), anti-PCNA (sc-56), anti-β-Actin (sc-47778) and anti-CK18 (sc-28264) were purchased from Santa Cruz. Densitometric measurement of the bands in Western blot analysis was performed using digitalized scientific software program using Kodak 2000R imaging system.

### Cell cycle analysis

Cells (70% confluent) were starved for 36 h to arrest them in G1 phase of the cell cycle, after which they were treated with 10 μM and 20 μM apigenin in RPMI 1640 complete media for 16 h. After treatment cells were collected, washed twice with chilled phosphate-buffered saline (PBS) and spun in a cold centrifuge at 600 *g* for 10 min. The pellet was fixed and resuspended in 50 μl PBS and 450 μl chilled methanol for 1 h at 4°C. The cells were washed twice with PBS at 600 *g* for 5 min and again suspended in 500 μl PBS and incubated with 5 ml RNase (20 μg/ml final concentration) for 30 min at 37°C. The cells were chilled over ice for 10 min and stained with propidium iodide (50 μg/ml final concentration) for 1 h and analyzed by flow cytometry and evaluated using Cell Quest & ModFit cell cycle analysis software.

### Wound healing migration assay

Cell migration was determined by means of wound-healing assay as previously described [55]. Cells were seeded into six-well plates and grown overnight. Then the cells were serum starved for 24 h. A sterile 200 μl pipette tip was used to scratch the cells to form a wound. The cells were washed with PBS and cultured in 10% fetal bovine serum medium with apigenin for 6 h and 16 h. Migration of the cells to the wound was visualized with an inverted Olympus phase-contrast microscope. The representative fields were photographed. The healing rate was quantified with measurements of the gap size after the culture using Image J software.

### Tumor xenograft study

The animal experiment was conducted in accordance with the guidelines established by the University's Animal Research Committee and with the NIH Guidelines for the Care and Use of Laboratory Animals. Approximately 1 million PC-3 and 22Rv1 cells suspended in 0.05 ml medium and mixed with 0.05 ml matrigel were subcutaneously injected in the left and right flank of each mouse to initiate tumor growth. After implantation, the animals were kept under supervision for growth of tumor. Two weeks after cell inoculation, animals were divided into three equal groups of six mice each. Apigenin (10 mg) was suspended in 1 ml vehicle material (0.5% methyl cellulose and 0.025% Tween 20) by sonication for 30 s at 4°C and further diluted for appropriate concentration. Apigenin, 20 and 50 μg/mouse/day was administered by gavage in 0.2 ml of a vehicle, daily for 8 weeks throughout the study. These doses are comparable to the daily consumption of flavonoid in humans as reported in previously published studies [34, 46].

### Apoptosis by ELISA

Apoptosis was assessed by M30-Apoptosense™ ELISA kit (Alexis Biochemicals, San Diego, CA) according to the manufacturer's protocol and color developed was read at 450-nm against the blank and values were expressed as fold change.

### Immunohistochemical analysis

Immunohistochemistry (IHC) for *p*-IKKα/β, PCNA and cleaved caspase-3 was performed on formalin-fixed, paraffin-embedded prostate tissue sections using a standard protocol as described previously using 3, 3′-diaminobenzidine and counterstaining with Mayer's hematoxylin [34]. Sections were examined with an inverted Olympus BX51 microscope and images were acquired with Olympus MicroSuite™ Five Software (Soft Imaging System, Lakewood, CO).

### Statistical analysis

The difference of relative density, kinase activity, IKKα/β phosphorylation and proliferation index between two groups was compared using *T*-test or paired *T*-test (for matched samples). For tumor volume and body weight, their trends over time were visualized by scatter plot (using mean ± SD against time, where SD stands for Standard Deviation of the Mean). The difference of tumor volume (mm^3^) and body weight, measured at the end of experiment (8 weeks after implantation of tumor), among three treatment groups (control, 20 μg and 50 μg apigenin) was examined using analysis of variance (ANOVA) followed by Turkey multiple comparison procedure. All tests are two-tailed and *p*-value less than 0.05 are considered to be statistically significant.

## SUPPLEMENTARY FIGURES LEGENDS


