# Supplementary material for: Apigenin blocks IKKα activation and suppresses prostate cancer progression
Source: Oncotarget. 2015 Sep 5;6(31):31216–32. doi: 10.18632/oncotarget.5157 (PMC4741599; doi:10.18632/oncotarget.5157)
Supplement: Supplementary file 1 [file oncotarget-06-31216-s001.pdf]

SUPPLEMENTARY FIGURES LEGENDS

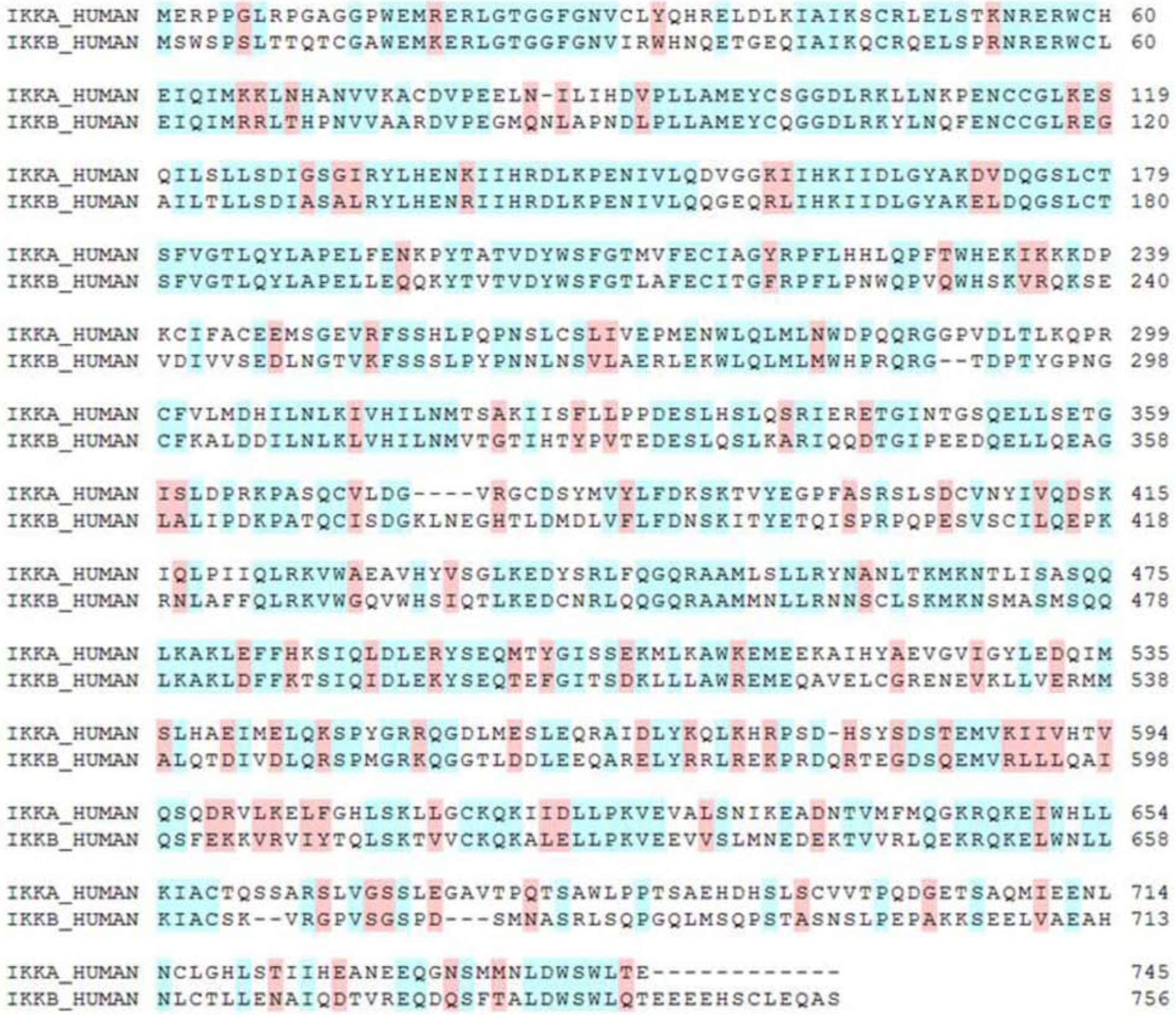

Supplementary Figure S1: Pairwise alignment of human IKKα and IKKβ protein sequences.

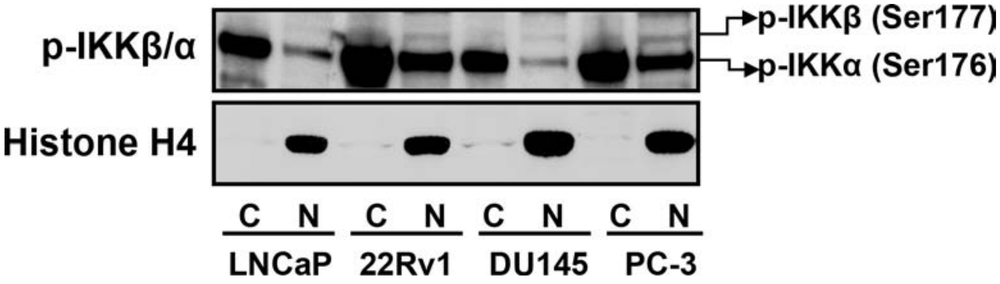

Supplementary Figure S2: Subcellular distribution of *p*-IKKα/β in human prostate cancer cells. Western blotting for *p*-IKKα/β protein expression in various human prostate cancer cells: LNCaP, 22Rv1, DU145 and PC-3 in the cytosolic and nuclear fractions. Prostate cancer 22Rv1 and PC-3 cells exhibited high *p*-IKKα/β expression in the nuclear fraction as well as in the cytosol, compared to LNCaP and DU145 cells. Histone H4 served as loading control. Details are described in 'materials and methods' section.

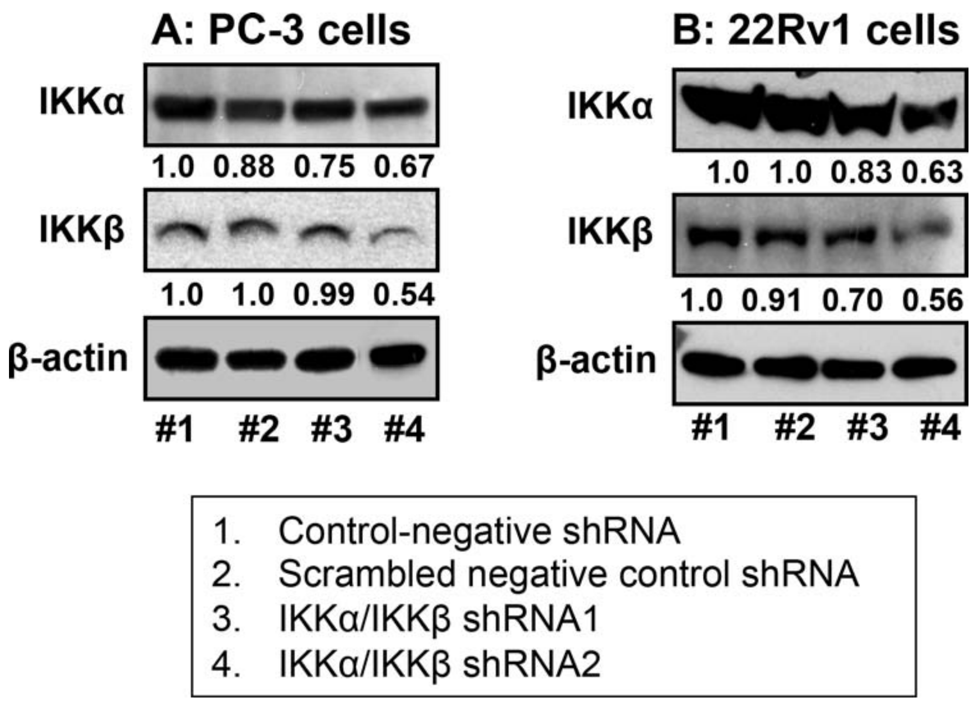

Supplementary Figure S3: Knockdown of IKKα and IKKβ in human prostate cancer cells. **A.** PC-3 and **B.** 22Rv1 cells were infected with a pool of viral particle containing 3 target specific IKKα and IKKβ constructs shRNA retroviral particle and one scrambled and one with negative shRNA, selected with polybrene for 15–20 passage and Western blotting was performed for IKKα and IKKβ. A significant decrease in IKKα and IKKβ protein expression by shRNA2 was observed in both cell lines used for cell cycle analysis. Lane 1, control-negative shRNA, Lane 2, scrambled negative control-shRNA, Lane 3, IKKα or IKKβ shRNA1 and Lane 4, IKKα or IKKβ shRNA2. β-Actin was used as loading control. Numeric values represent the protein level normalized to the loading control (actin). Details are described in ‘materials and methods’ section.

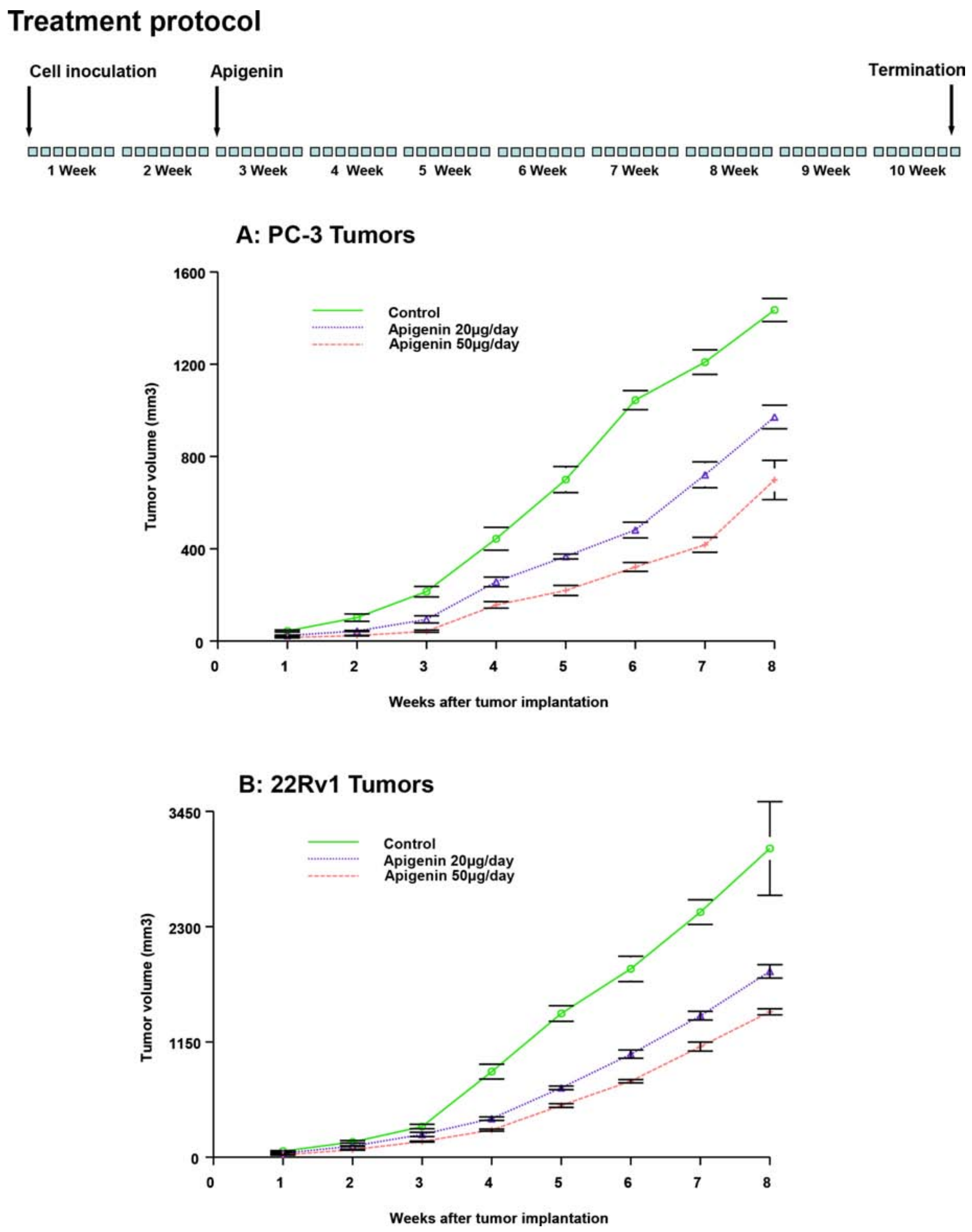

Supplementary Figure S4: Effect of apigenin on prostate tumor growth inhibition in athymic nude mouse xenograft. A. PC-3 and **B.** 22Rv1 tumors. Approximately 1 million cells were injected into both flanks of each mouse to initiate prostate tumor xenograft, and apigenin was provided to the animals 2 week after cell inoculation. Mice were fed ad libitum with Teklad 8760 autoclaved high-protein diet. Apigenin was provided with 0.5% methylcellulose and 0.025% Tween-20 as vehicle to these animals perorally on a daily basis. Group I, control, received 0.2 mL vehicle only, II group received 20 µg apigenin per mouse in 0.2 mL vehicle, and III group received 50 µg apigenin per mouse in 0.2 mL vehicle daily for 8 week and experiment was terminated. Once the tumor xenografts started growing, their sizes were measured by volume twice weekly in two dimensions throughout the study. Values are Mean ± SE, $n = 6–8$, repeated twice with similar results. $^{**}P < 0.001$, compared to vehicle treated control.

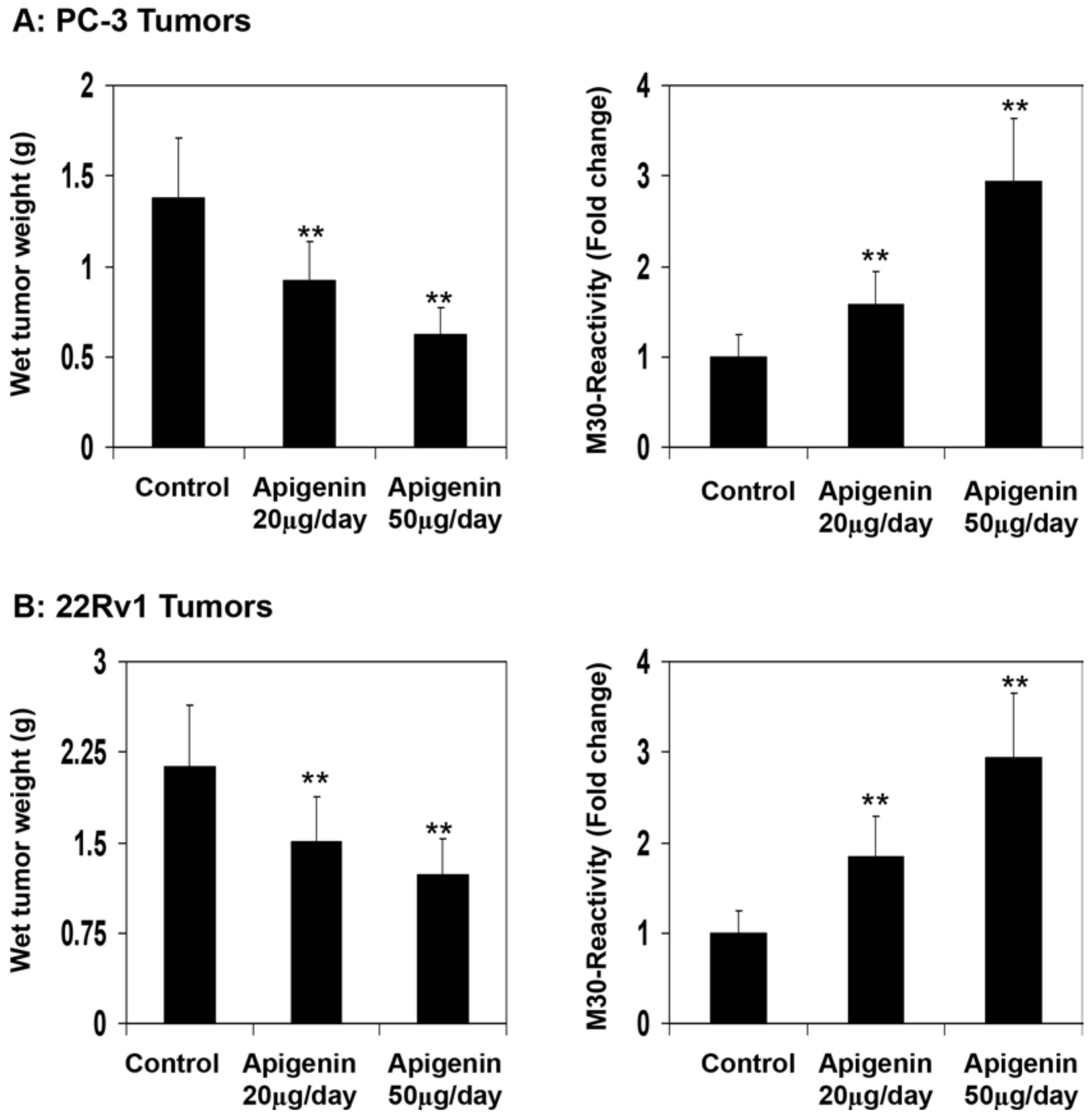

Supplementary Figure S5: Effect of apigenin on prostate tumor weight and induction of apoptosis in athymic nude mouse xenograft. A. PC-3 and **B.** 22Rv1 tumors obtained after tumor implantation and feeding mice with 20- and 50- µg apigenin in 0.2 ml vehicle daily for 8 weeks. Details are described in Supplemental figure 4. Wet weight of tumors is represented as the mean of 6–8 tumors from each group and quantitative measurement of apoptosis as demonstrated by M30 reactivity in PC-3 and 22Rv1 tumors after apigenin intake at the indicated doses. Values are Mean ± SD, $n = 6–8$, repeated twice with similar results. $^{**}P < 0.001$, compared to vehicle treated control.
